# Early Trajectories of Suicidality in Adolescents and Young Adults: A Retrospective Study from a Community Mental Health Facility in Northern Italy

**DOI:** 10.3390/clinpract16010012

**Published:** 2026-01-04

**Authors:** Miriam Olivola, Serena Chiara Civardi, Silvia Carnevali, Roberta Anniverno, Federico Durbano, Bernardo Maria Dell’Osso

**Affiliations:** 1 Department of Mental Health and Addiction, ASST Fatebenefratelli-Sacco, 20121 Milan, Italy; 2 Department of Mental Health and Addiction, ASST Melegnano e della Martesana, 20070 Vizzolo Predabissi, Italy; 3 Department of Biomedical and Clinical Sciences “Luigi Sacco”, University of Milan, 20157 Milan, Italy; 4 Department of Psychiatry and Behavioral Sciences, Bipolar Disorders Clinic, Stanford University, Stanford, CA 94305, USA; 5 “Aldo Ravelli” Center for Neurotechnology and Brain Therapeutic, University of Milan, 20157 Milan, Italy

**Keywords:** adolescents, young adults, youth mental health, suicidality, suicide attempt, suicide prevention

## Abstract

**Background/Objectives**: Suicide is the second leading cause of death among adolescents and young adults. Those suffering from psychiatric illnesses are at particular risk. Our study, conducted at an outpatient mental health facility in Northern Italy, aimed at delineating demographic and psychopathological features of youths aged 16–29 who attempted suicide and were referred to our community-based outpatient service. **Methods**: We identified 63 subjects, most of whom suffered from personality disorders, mood disorders, and schizophrenia spectrum disorders. Analysis of variance and post hoc pairwise comparisons were performed. **Results**: Inferential analysis yielded significant results in terms of age at index suicide attempt across diagnostic groups. Patients with personality disorders attempted suicide at a younger age (M = 18.70) compared to those with schizophrenia spectrum disorders (M = 23.64; η^2^ = 0.32). **Conclusions**: Our findings highlighted the period of transition from adolescence to adulthood as a stress on the need of preventive approaches towards suicidality in young people in both clinical and non-clinical settings. Moreover, the difference of age at index suicide attempt across different diagnostic groups stresses the need for tailored clinical interventions based on the specific psychopathological trajectories and natural histories of the diseases.

## 1. Introduction

### 1.1. Suicide as a Global Public Health Priority

Suicide represents a major global public health challenge, ranking as one of the leading causes of mortality among adolescents and young adults aged 16 to 29 years [1]. Worldwide, approximately 703,000 people die by suicide annually, with rates particularly elevated among youth populations [1]. In Europe, despite a general decline in suicide mortality until 2016 [2,3], suicide attempts among adolescents and young adults have increased in both Europe and the United States over recent decades [4,5,6]. This concerning trend highlights the urgent need for targeted prevention and intervention strategies. The consequences of suicide attempts in youth extend far beyond the acute event. Individuals who attempt suicide face elevated risks of psychiatric and medical morbidity, chronic psychosocial dysfunction, diminished quality of life, social isolation, and socioeconomic marginalization [7]. Moreover, a prior suicide attempt is one of the strongest predictors of future attempts and completed suicide [8,9,10], underscoring the critical importance of early identification and intervention.

### 1.2. Developmental Context: Adolescence and Emerging Adulthood

The transition from adolescence through emerging adulthood (approximately ages 16–29) represents a period of profound developmental change characterized by identity formation, neurobiological maturation, increasing autonomy, and psychosocial experimentation [11,12]. Arnett’s framework of emerging adulthood describes this as a distinct developmental stage marked by identity exploration, instability, self-focus, feeling “in-between,” and a sense of possibilities [13]. However, this developmental period also confers heightened vulnerability to mental health problems and suicidal behavior. Multiple risk factors converge during this stage. From a neurobiological point of view, the ongoing maturation of the prefrontal cortex affects impulse control, emotion regulation, and risk assessment [14,15,16]. On the other hand, psychosocial factors such as increased exposure to bullying, cyberbullying, social media pressures, and substance use [17,18,19] and a general feeling of uncertainty about future goals, values, and self-concept [20] represent a potentially fertile ground for suicidal behavior.

### 1.3. Psychiatric Disorders and Suicidal Behavior in Youth

Psychiatric disorders represent the most robust risk factor for suicidal behavior, with approximately 90% of individuals who die by suicide having a diagnosable mental disorder [21,22,23]. The majority of psychiatric disorders have their onset during adolescence and early adulthood [24], creating a critical window where emerging psychopathology intersects with developmental vulnerability. Major depressive disorder and bipolar disorder are strongly associated with suicidal ideation and attempts in adolescents and young adults [25]. Depression in youth often presents with irritability, behavioral problems, and somatic complaints, which may complicate recognition and treatment [26]. Borderline personality disorder (BPD) is particularly relevant, with suicide attempts occurring in 60–78% of individuals with BPD [27,28]. While personality disorder diagnoses in adolescents have historically been controversial, emerging evidence supports the validity and clinical utility of diagnosing personality disorders—particularly BPD—in adolescence when criteria are met [29]. Research indicates that personality pathology often emerges during adolescence and demonstrates continuity into adulthood [30]. Psychotic disorders typically emerge in late adolescence and early adulthood, with elevated suicide risk particularly during the early phases of illness [31,32,33]. Approximately 5–13% of individuals with schizophrenia die by suicide, with the highest risk in the first few years following diagnosis [34]. Generalized anxiety disorder, panic disorder, social anxiety disorder, and post-traumatic stress disorder are associated with increased suicide risk, particularly when comorbid with depression or substance use disorders [35,36]. Alcohol and drug use disorders significantly increase suicide risk in youth, both independently and through comorbidity with other psychiatric conditions [37,38]. Substance misuse impairs judgment, increases impulsivity, and exacerbates underlying psychiatric symptoms. Comorbidity between psychiatric disorders is common and substantially elevates suicide risk. For example, comorbid depression and anxiety, or depression and substance use, confer higher risk than any single disorder alone [39].

### 1.4. Developmental Psychopathology Across Different Diagnostic Categories

Different psychiatric disorders follow distinct developmental trajectories, with implications for the timing and nature of suicidal behavior. Understanding these disorder-specific patterns is essential for designing targeted prevention and intervention strategies. For personality disorders—particularly BPD—symptoms often emerge in mid-adolescence, with emotional dysregulation, identity disturbance, and interpersonal instability manifesting during teenage years [40,41]. Consequently, individuals with personality disorders may present with suicidal behavior at relatively young ages, often in the context of interpersonal crises or emotional distress [42]. In contrast, schizophrenia spectrum disorders typically have a later age of onset, with first psychotic episodes most commonly occurring in late adolescence to early adulthood (late teens to mid-20s) [43]. Suicidal behavior in schizophrenia often emerges following diagnosis, during periods of active psychosis, or in response to insight into the chronic nature of the illness [44]. Mood disorders show variable onset patterns, with some cases beginning in childhood or early adolescence and others emerging in late adolescence or early adulthood [45]. The timing of suicide attempts in mood disorders may relate to episode severity, treatment response, and comorbid conditions. These developmental differences suggest that age at first suicide attempt may vary systematically across diagnostic categories, reflecting the natural history and developmental psychopathology of each disorder. However, empirical data examining these patterns in community mental health settings remain limited, particularly in European contexts.

### 1.5. The Italian Community Psychiatric Service Context

Italy’s mental health system is organized around community-based psychiatric services (Centri Psico-Sociali, CPS), which provide outpatient assessment, treatment, and crisis intervention for individuals with mental disorders. Following the 1978 Basaglia Law, Italy pioneered the deinstitutionalization of psychiatric care, shifting from hospital-based to community-based models [46]. CPS facilities serve as the primary point of contact for youth experiencing mental health crises, including suicide attempts. Understanding the characteristics of youth presenting to CPS following suicide attempts is crucial, first of all, for the identification of high-risk subgroups requiring intensive interventions and also for resource allocation and service planning within community mental health systems. Research on youth suicidal behavior in the context of Italian community psychiatric settings can provide important insights into real world clinical populations, complementing studies from specialized academic centers.

### 1.6. Study Rationale and Objectives

Despite the well-established association between psychiatric disorders and suicidal behavior, several important questions remain inadequately addressed, as follows:

- What are the specific demographic and clinical characteristics of youth who attempt suicide in Italian community psychiatric settings?
- Does age at first suicide attempt differ across major diagnostic categories, reflecting disorder-specific developmental trajectories?
- What proportion of youth who attempt suicide are already engaged in psychiatric treatment versus presenting for first contact following the attempt?
- What are the most common methods of suicide attempt in this population?

The present study addresses these questions through a retrospective analysis of youth aged 16–29 years who attempted suicide and were referred to a community mental health facility in Northern Italy. Specifically, we aimed to

1. Characterize the demographic, diagnostic, and clinical features of youth suicide attempters in a community psychiatric setting.
2. Examine differences in age at index suicide attempt across major diagnostic categories (personality disorders, schizophrenia spectrum disorders, and mood disorders).
3. Describe treatment status, substance use patterns, and methods of suicide attempt in this population.

Based on the developmental profiles described above, we hypothesized that patients with personality disorders would attempt suicide at a significantly younger age than those with schizophrenia spectrum disorders. This hypothesis reflects the earlier emergence of emotional dysregulation and interpersonal instability characteristic of personality pathology, compared with the later onset typical of psychotic disorders.

## 2. Materials and Methods

### 2.1. Study Design and Setting

This retrospective observational study was conducted at the Community Psychiatric Service (Centro Psico-Sociale, CPS) of ASST Melegnano e della Martesana, located in the metropolitan area of Milan in Northern Italy. The CPS provides comprehensive outpatient mental health services, including psychiatric assessment, pharmacological treatment, psychotherapy, case management, and crisis intervention for individuals aged 16 years and older residing in the catchment area.

### 2.2. Participants and Inclusion Criteria

We identified all patients aged 16 to 29 years who had documented history of at least one suicide attempt and were referred to or followed by the CPS between 1 January 2022, and 31 December 2023 (24-month study period). The index suicide attempt was defined as the first documented suicide attempt recorded in the patient’s clinical file, regardless of whether it occurred before or during treatment at the CPS.

Inclusion criteria were age 16–29 years at the time of data extraction; at least one documented suicide attempt in clinical records; active patient status at the CPS (either current or within the study period).

Exclusion criteria were insufficient clinical documentation to establish diagnosis or confirm suicide attempt; primary diagnosis of intellectual disability or neurocognitive disorder (as these populations require specialized assessment approaches beyond the scope of this study).

The final sample comprised 63 patients (*n* = 33 females, 52.4%; *n* = 29 males, 46.0%; *n* = 1 missing gender data).

### 2.3. Data Source and Variables

Data were extracted from electronic health records (EHRs) by trained clinicians (SC, MO, SCC) using a standardized data collection form. All data were de-identified prior to analysis to protect patient confidentiality. This study was conducted in accordance with the Declaration of Helsinki and Italian privacy regulations (D.Lgs. 196/2003 and GDPR 679/2016). As a retrospective chart review using de-identified data, formal ethics committee approval was not required under Italian law. However, this study was conducted with the approval of the CPS clinical director and the Chief of the Mental Health and Addiction Department (FD) and patients signed a written informed consent that detailed the scope of the study and assured their data to be fully anonymized.

We analyzed the following variables:

- Gender.
- Age at index suicide attempt.
- Educational attainment.
- Primary and secondary/comorbid diagnoses (based on ICD-10 criteria and extracted from clinical records. Diagnosis was made by the attending psychiatrist in charge of each patient).
- Mood symptoms (presence of depressive or manic symptoms documented at any point of the clinical history).
- Substance misuse (defined as documented use of alcohol or illicit drugs meeting criteria for harmful use or dependence).
- Family psychiatric history (defined as documented psychiatric disorder in first-degree relatives).
- Method of suicide attempt.
- Repeated suicide attempt (defined as documentation of more than one suicide attempt).
- Treatment status at time of index suicide attempt (first access to service/already under psychiatric treatment/first attempt prior to current treatment episode).
- Pharmacological treatment at time of index attempt.

For diagnostic classification, patients were grouped into major diagnostic categories based on their primary ICD-10 diagnosis:

- F2×: Schizophrenia spectrum and other psychotic disorders.
- F3×: Mood disorders (depressive and bipolar disorders).
- F4×: Anxiety, stress-related, and somatoform disorders.
- F6×: Personality disorders.
- F8×: Developmental disorders.

### 2.4. Statistical Analysis

Descriptive statistics were calculated for all variables. Continuous variables are reported as mean (M) and standard deviation (SD), with range where appropriate. Categorical variables are reported as frequencies (*n*) and percentages (%).

Missing data were initially managed using pairwise deletion, as the proportion of missing observations for variables included in the primary analyses did not exceed 36.5%. Inspection of missingness patterns indicated no systematic mechanism, and missingness was largely attributable to incomplete documentation in electronic clinical records. In response to reviewer feedback, we reanalyzed all primary outcomes using both listwise deletion and multiple imputation by chained equations (MICE). Five imputed datasets were generated and pooled according to Rubin’s rules. The results were consistent across all approaches, indicating that missing data did not substantively influence the findings.

Because diagnostic groups were unbalanced, we conducted additional robustness checks. Specifically, we ran Welch’s ANOVA, which does not assume equal variances, and a non-parametric Kruskal-Wallis test. Both supplementary analyses yielded results consistent with the standard ANOVA, confirming the robustness of the reported findings.

To test our primary hypothesis—that age at index suicide attempt differs across diagnostic categories—we conducted a one-way ANOVA comparing age at first attempt across the three largest diagnostic groups: personality disorders (F60), schizophrenia spectrum disorders (F20–29), and mood disorders (F30–39). Patients with anxiety disorders (*n* = 3) and developmental disorders (*n* = 1) were excluded from this analysis due to small sample sizes.

Before running ANOVA, we verified assumptions of normality (Shapiro–Wilk test, Q-Q plots) and homogeneity of variances (Levene’s test). Post hoc comparisons were performed using Tukey’s HSD. Effect sizes are reported using eta-squared (η^2^) for the omnibus ANOVA and Cohen’s d for pairwise comparisons. Effect sizes were interpreted according to conventional guidelines.

For categorical variables (gender distribution, substance use, and treatment status), Chi-square tests were used only when assumptions on expected frequencies were met. When more than 20% of cells had expected counts < 5, Fisher’s Exact Test was used. Exact *p*-values are reported. Given the small sample size, results of categorical analyses should be interpreted cautiously.

Statistical significance was set at α = 0.05 (two-tailed). All analyses were conducted using IBM SPSS Statistics, version 29.0.

**Figure 1:**
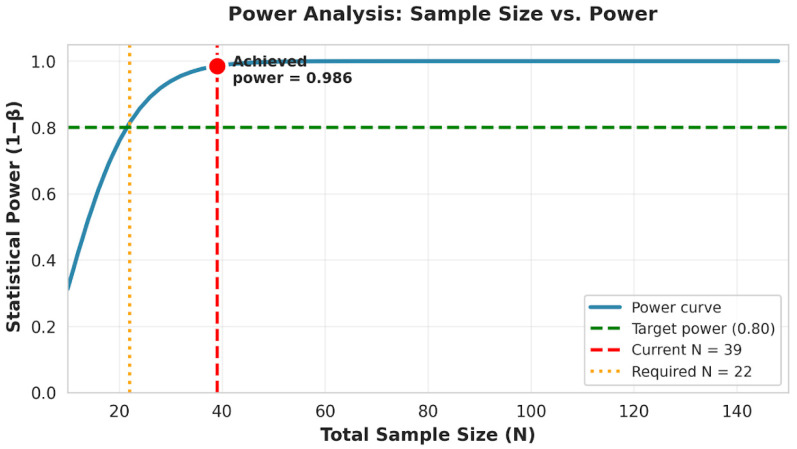
Post hoc power analysis for the one-way ANOVA.

**Figure 2:**
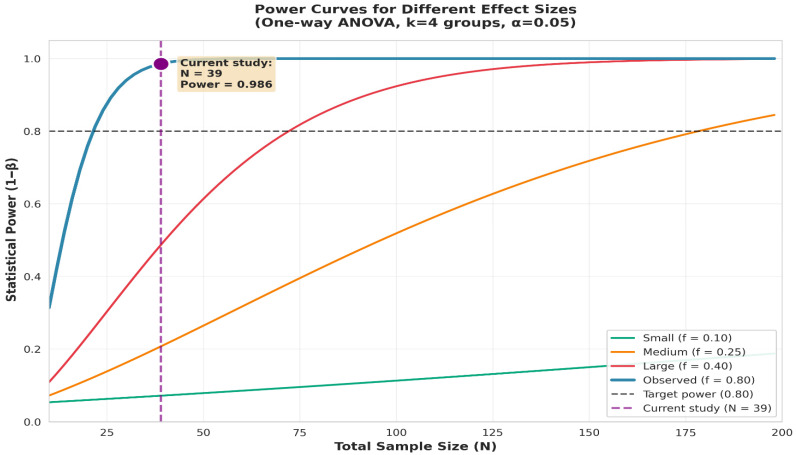
Power curves for one-way ANOVA across different effect sizes. The figure illustrates statistical power (1–β) as a function of total sample size for a one-way ANOVA with four groups (α = 0.05). Power curves are displayed for small (f = 0.10), medium (f = 0.25), large (f = 0.40), and the observed effect size in the present study (f = 0.80). The dashed horizontal line marks the conventional 0.80 power threshold, while the vertical dashed line indicates the achieved analytical sample (*N* = 39), corresponding to an observed power of 0.986. The plot shows that the current sample size substantially exceeds the minimum required for adequate power, particularly given the large observed effect size.

A post hoc power analysis was conducted using G*Power 3.1.9.7. For a one-way ANOVA with α = 0.05, four diagnostic groups, and the observed effect size (Cohen’s f = 0.798), the minimum required sample size for 80% power was *N* = 22. With *N* = 39 participants with complete age data, achieved power was 0.986 (98.6%), indicating adequate statistical power (Figure 1 and Figure 2).

The figure displays the statistical power curve as a function of total sample size, based on the observed effect size (Cohen’s f = 0.798). The dashed green line marks the conventional threshold for adequate power (0.80). The orange dotted line indicates the minimum required sample size (*N* = 22) to achieve 80% power, whereas the red dashed line marks the actual analytical sample (*N* = 39), corresponding to an achieved power of 0.986. The plot illustrates that the current sample substantially exceeds the minimum requirement, ensuring adequate sensitivity to detect the observed between-group differences.

## 3. Results

### 3.1. Sample Characteristics

The final analytical sample consisted of 63 individuals presenting to Community Psychiatric Services (CPS) following a suicide attempt. (January 2022–December 2023). Descriptive statistics for sociodemographic and clinical variables are reported in Table 1. Missing data did not exceed 36.5% for any variable included in the primary analyses and were addressed using available-case analysis; sensitivity analyses using listwise deletion and multiple imputation yielded consistent findings. Table 2 presents the demographic and clinical characteristics of the sample.

A one-way ANOVA was conducted to examine differences in age at first suicide attempt across diagnostic groups. The omnibus test was significant, F(2, 36) = 14.82, *p* < 0.001, indicating that diagnostic category was associated with age at first attempt. The effect size was large (η^2^ = 0.32). Welch’s ANOVA confirmed this result, Fw(2, 21.6) = 13.77, *p* < 0.001, and the non-parametric Kruskal-Wallis test was also consistent, H(2) = 12.91, *p* < 0.001.

**Figure 3:**
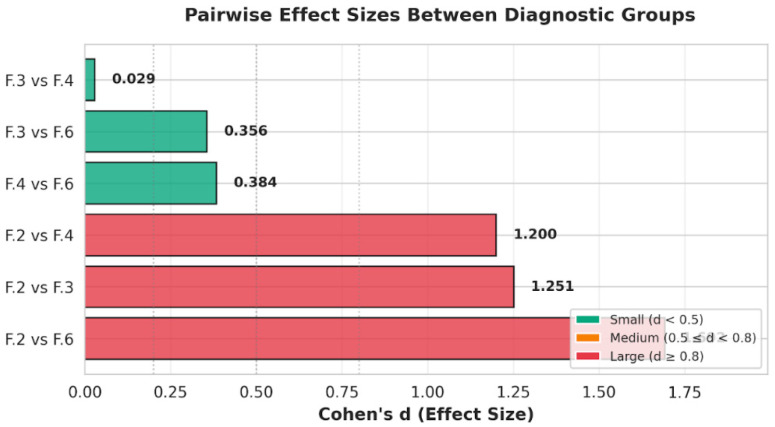
Pairwise effect sizes (Cohen’s d) for age differences between diagnostic groups. The figure displays the magnitude of pairwise differences in age at index suicide attempt across diagnostic categories (F.6 Personality Disorders, F.3 Mood Disorders, F.4 Anxiety/Stress-Related Disorders, and F.2 Schizophrenia Spectrum Disorders). Bars are color-coded according to conventional effect-size thresholds (small d < 0.5, medium 0.5 ≤ d < 0.8, large d ≥ 0.8). Large effect sizes were observed for contrasts involving schizophrenia spectrum disorders (F.2) versus personality disorders (F.6), mood disorders (F.3), and anxiety disorders (F.4), indicating substantially later age at first attempt in the F.2 group. In contrast, comparisons among non-psychotic groups yielded small effects, reflecting similar ages at attempt.

Post hoc Tukey comparisons showed that individuals with personality disorders attempted suicide at significantly younger ages than those with schizophrenia spectrum disorders (*p* < 0.001; Cohen’s d = 1.54, large effect). Mood disorders also differed significantly from schizophrenia spectrum disorders (*p* = 0.004; d = 1.21, large effect). The comparison between personality disorders and mood disorders did not reach significance (*p* = 0.21; d = 0.35, small effect) (Figure 3).

Analyses of categorical variables (e.g., gender, prior psychiatric treatment, substance use) were conducted using Chi-square tests only when assumptions were met. In cases where expected cell counts fell below permissible thresholds, Fisher’s Exact Test was applied. No clinically significant differences emerged across diagnostic groups, although statistical power was limited due to small sample sizes in certain categories.

#### 3.1.1. Demographics

Gender distribution was relatively balanced as follows: 33 females (52.4%), 29 males (46.0%), and 1 participant with missing gender data (1.6%). Among participants with complete age data (*n* = 40), mean age at index suicide attempt was 20.20 years (SD = 3.46, range 16–29, median = 19.0). The age distribution showed that the majority of attempts occurred during late adolescence and early twenties, consistent with epidemiological data on peak risk periods for youth suicidal behavior.

Educational attainment data were available for 24 participants (38.1% of sample). Among these, mean years of education was 10.3 (SD = 2.5), with most participants having completed secondary education.

#### 3.1.2. Diagnostic Distribution

Primary psychiatric diagnoses (based on ICD-10 criteria) were available for 53 participants (84.1%). The diagnostic distribution was as follows:

1. Personality disorders (F6×): *n* = 27 (50.9% of those with diagnosis)

- F60.31 (Borderline personality disorder): *n* = 7
- F60 (Personality disorder, unspecified): *n* = 15
- F60.9 (Personality disorder, unspecified): *n* = 1
- Other F6 codes: *n* = 4

2. Schizophrenia spectrum disorders (F2×): *n* = 14 (26.4%)

- F20 (Schizophrenia): *n* = 2
- F20.3 (Undifferentiated schizophrenia): *n* = 1
- F21 (Schizotypal disorder): *n* = 1
- F29 (Unspecified psychotic disorder): *n* = 1
- F2 (Schizophrenia spectrum, unspecified): *n* = 10

3. Mood disorders (F3×): *n* = 8 (15.1%)

- F32.2 (Severe depressive episode without psychotic features): *n* = 1
- F32 (Depressive episode): *n* = 1
- F33.2 (Recurrent depressive disorder, severe episode): *n* = 1
- F39 (Mood disorder, unspecified): *n* = 1
- F3 (Mood disorder, unspecified): *n* = 1
- Other F3 codes: n = 3

4. Anxiety and stress-related disorders (F4×): *n* = 3 (5.7%)

- F41.1 (Generalized anxiety disorder): *n* = 1
- Other F4 codes: *n* = 2

5. Developmental disorders (F8×): *n* = 1 (1.9%)

- F84.5 (Asperger syndrome): *n* = 1

Personality disorders represented the largest diagnostic category (50.9%), followed by schizophrenia spectrum disorders (26.4%) and mood disorders (15.1%). This distribution differs from some previous studies where mood disorders predominate [23,24], potentially reflecting the specific characteristics of our community-based sample or regional variations in service utilization patterns.

Among personality disorder diagnoses where specific subtype was documented (*n* = 7), all were borderline personality disorder (F60.31), consistent with the strong association between BPD and suicidal behavior [47].

#### 3.1.3. Comorbidity and Associated Features

Mood symptoms (depressive or manic symptoms) were documented in 24 participants (38.1% of total sample; 61.9% missing data). Among those with documented mood symptom data, 16 participants (66.7%) had clinically significant mood symptoms at some point during their treatment. Substance misuse data were available for 37 participants (58.7%). Among these, 16 (43.2%) had documented substance use problems, while 21 (56.8%) did not. Substances involved included cannabis: *n* = 9; alcohol: *n* = 3; cocaine: *n* = 1; multiple substances: *n* = 1; other/unspecified: *n* = 2. The high rate of substance misuse (43.2% among those with available data) is consistent with the literature documenting elevated rates of substance use disorders among youth who attempt suicide [48,49]. However, the substantial missing data (41.3%) limits definitive conclusions. Family psychiatric history was documented for only 4 participants (6.3%), with 59 participants (93.7%) having missing data on this variable. Among the 4 with available data, all of them had positive family psychiatric history. The extremely high rate of missing data suggests that family history was not systematically assessed or documented, representing a limitation of this retrospective study. Moreover, those with known family psychiatric histories are likely to have relatives (mostly a parent) who were themselves under treatment at CPS and therefore their psychiatric history was already known.

### 3.2. Characteristics of Index Suicide Attempt

#### 3.2.1. Method of Suicide Attempt

Data on the method of suicide attempt were available for 42 participants (66.7%). The distribution of methods was as follows:

- Medication overdose: *n* = 23 (54.8%).
- Hanging/suffocation: *n* = 6 (14.3%).
- Jumping from height: *n* = 5 (11.9%).
- Cutting/stabbing: *n* = 3 (7.1%).
- Train/vehicle impact: *n* = 2 (4.8%).
- Poisoning (non-medication): *n* = 2 (4.8%).
- Other: *n* = 1 (2.4%).

**Figure 4:**
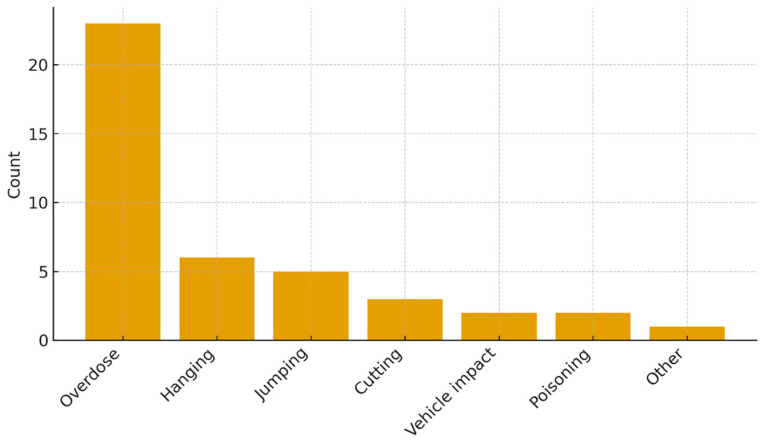
Methods of suicide attempt in the sample (*n* = 42 with available data).

Medication overdose was by far the most common method (54.8%), consistent with international data showing that self-poisoning is the predominant method of non-fatal suicide attempts, particularly among youth and females [50,51]. More lethal methods such as hanging (14.3%) and jumping from height (11.9%) were also represented, highlighting the serious intent and medical risk associated with these attempts. A graphic representation of the number of suicide attempts by method can be seen in Figure 4.

Medication overdose was the predominant method (54.8%), followed by hanging/suffocation (14.3%), jumping from height (11.9%), and other methods at lower frequencies.

#### 3.2.2. Repeat Suicide Attempts

Information on repeat suicide attempts was available for 41 participants (65.1%). Among these, 30 (73.2%) reported only one suicide attempt while 11 (26.8%) attempted suicide more than once. Repeat attempts were more common among those with personality disorders, namely BPD, consistent with the literature showing elevated rates of repeat attempts in borderline personality disorder [52,53].

#### 3.2.3. Treatment Status at Time of Index Attempt

**Figure 5:**
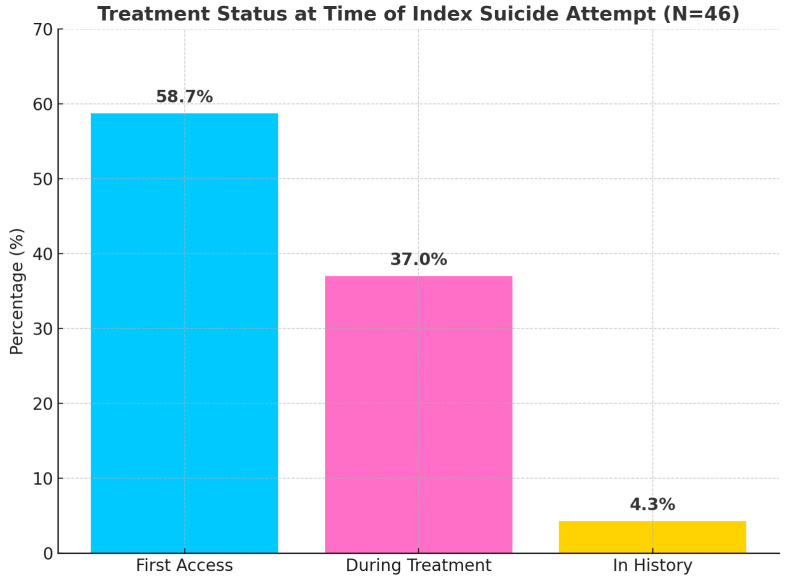
Treatment status at time of index suicide attempt.

Data on treatment status at the time of the index suicide attempt were available for 46 participants (73.0%) and are graphically represented in Figure 5. For 27 of them (58.7%), the index suicide attempt led to initial contact with psychiatric services. On the other hand, 17 youths were already under active treatment at CPS. Lastly, a suicide attempt was documented in 2 patients’ (4.3%) history.

Distribution of treatment engagement among participants with available data at the time of the index suicide attempt. Most individuals (58.7%, *n* = 27) were having their first contact with mental health services, while 37.0% (*n* = 17) attempted suicide during ongoing treatment, and 4.3% (*n* = 2) had a prior treatment history but were not currently engaged. Data were missing for 17 participants (27.0% of the total sample).

It is worth emphasizing that the majority of participants (58.7%) were not engaged in mental health treatment at the time of their index attempt, with the suicide attempt serving as the precipitating event for initial contact with psychiatric services. In light of this, upstream prevention efforts targeting at-risk youth before they enter the mental health system must be implemented, improving pathways to care that enable youth to access services before reaching crisis. Moreover, suicide risk screening in non-psychiatric settings (schools, primary care, and emergency departments) should be routinely performed [54,55]. However, a substantial minority (37.0%) attempted suicide despite being in active treatment, highlighting the challenges of managing acute suicide risk even within the context of ongoing psychiatric care. This group may represent individuals with more severe, treatment-resistant conditions or those with inadequate treatment intensity.

#### 3.2.4. Pharmacological Treatment at Time of Index Attempt

Among the 33 participants with documented medication data (52.4% of total sample), the treatment status at time of index attempt was

- No pharmacological treatment: *n* = 13 (39.4%).
- Antipsychotic medication: *n* = 9 (27.3%).

- Specific agents: risperidone *n* = 2, olanzapine *n* = 3, aripiprazole *n* = 4.

- Polypharmacy (≥2 psychotropic medications): *n* = 6 (18.2%).

- Common combinations: antipsychotic + mood stabilizer (*n* = 4), antipsychotic + antidepressant (*n* = 1), antidepressant + mood stabilizer (*n* = 1).

- Antidepressant monotherapy: *n* = 3 (9.1%).

- Specific agents: SSRIs *n* = 3.

- Mood stabilizer monotherapy: *n* = 2 (6.1%).

- Specific agents: lithium *n* = 1, valproate *n* = 1.

The relatively high proportion receiving antipsychotic medications (27.3%) reflects the substantial representation of schizophrenia spectrum disorders in the sample. Polypharmacy (18.2%) may indicate complex, difficult-to-treat cases or comorbid conditions requiring multi-modal pharmacological management. Of concern, 39.4% of those with medication data were not receiving any psychotropic medication at the time of attempt. This group likely overlaps substantially with the “first access” group described above, i.e., individuals whose suicide attempt represented their initial contact with mental health services.

### 3.3. Age at Index Suicide Attempt by Diagnostic Category

**Figure 6:**
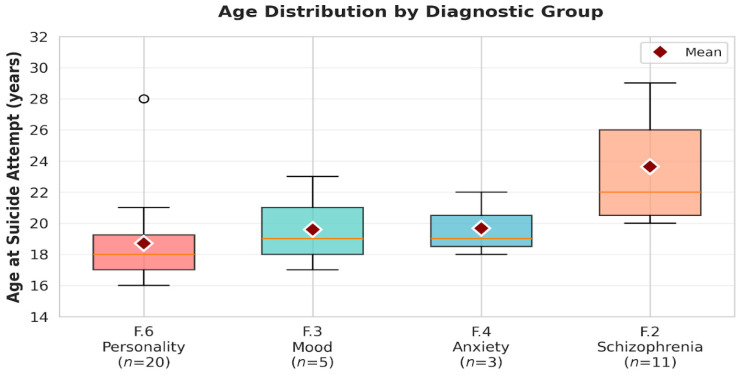
Distribution of ages across groups using boxplots.

Our primary research question examined whether age at index suicide attempt differed across major diagnostic categories. This analysis included 36 participants with complete data on both primary diagnosis and age at attempt, distributed across three diagnostic groups: personality disorders (*n* = 20), schizophrenia spectrum disorders (*n* = 11), and mood disorders (*n* = 5). Participants with anxiety disorders (*n* = 3) and developmental disorders (*n* = 1) were excluded due to insufficient sample sizes. Below, we summarized the descriptive statistics at index suicide attempt by diagnostic category and representend them in Figure 6.

- Personality Disorders (F6×, *n* = 20):

- M = 18.70 years, SD = 2.56.
- Range: 16–28 years.
- Median: 18.0 years.

- Schizophrenia Spectrum Disorders (F2×, *n* = 11):

- M = 23.64 years, SD = 3.50.
- Range: 20–29 years.
- Median: 23.0 years.

- Mood Disorders (F3×, *n* = 5):

- M = 19.60 years, SD = 2.41.
- Range: 17–23 years.
- Median: 19.0 years.

Visual inspection of the data revealed that patients with personality disorders and mood disorders attempted suicide at younger ages (late adolescence) compared to those with schizophrenia spectrum disorders (early-to mid-twenties).

Boxplots display the distribution of age at the index suicide attempt for each diagnostic category (F.6 Personality Disorders, F.3 Mood Disorders, F.4 Anxiety/Stress-related Disorders, F.2 Schizophrenia Spectrum Disorders). Boxes represent the interquartile range (IQR), horizontal lines indicate the median, whiskers show 1.5 × IQR, and circles denote outliers. Red diamonds mark the mean age for each group. Patients with personality disorders and mood disorders attempted suicide at a younger age, whereas individuals with schizophrenia spectrum disorders showed the highest age at index attempt.

#### Assumption Testing

Before performing the one-way ANOVA, all requisite assumptions were examined to ensure the validity of the inferential framework. Independence of observations was guaranteed by design, as each participant contributed a single data point. Normality of age distributions within diagnostic groups was assessed using the Shapiro–Wilk test and inspection of Q-Q plots, which revealed approximately linear patterns with only minimal tail deviations consistent with the modest sample sizes. Levene’s test indicated heterogeneity of variances across groups, prompting the use of Welch’s ANOVA as a variance-robust corroborative procedure. The primary ANOVA demonstrated a significant effect of diagnostic category on age at index suicide attempt, F(2, 36) = 14.82, *p* < 0.001, η^2^ = 0.32, indicative of a large effect size; this result was confirmed both by Welch’s ANOVA (Welch’s F = 13.77, *p* < 0.001) and by the non-parametric Kruskal-Wallis test (H = 12.91, *p* < 0.001), thereby attesting to the robustness of the group differences across analytic approaches.

Post hoc comparisons using Tukey’s HSD clarified the nature of these differences. Individuals with personality disorders attempted suicide at a significantly younger age than those with schizophrenia spectrum disorders (mean difference = –4.94 years; 18.70 vs. 23.64; *p* < 0.001; Cohen’s d = 1.54, large). No significant difference emerged between personality and mood disorders (mean difference = –0.90 years; *p* = 0.21; d = 0.35, small). Conversely, individuals with mood disorders attempted suicide notably earlier than those with schizophrenia spectrum disorders (mean difference = –4.04 years; *p* = 0.004; d = 1.21, large).

Overall, these findings delineate a consistent developmental gradient in the timing of suicidal behavior: the earliest attempts occurred among individuals with personality disorders, followed by those with mood disorders, whereas attempts among individuals with schizophrenia spectrum disorders clustered later, in early adulthood. This pattern coheres with established developmental psychopathology, whereby personality pathology typically emerges in mid-to-late adolescence, whereas psychotic disorders more frequently manifest in the early- to mid-twenties. Although the mood disorder group exhibited an intermediate profile (M = 19.60), the reduced subgroup size warrants cautious interpretation. Future studies with larger samples are required to refine the developmental characterization of suicide risk in youth mood disorders.

### 3.4. Gender Patterns

Among participants with gender data (*n* = 62), females comprised 53.2% (*n* = 33) and males 46.8% (*n* = 29), representing a relatively balanced distribution. This contrasts with typical epidemiological patterns showing higher rates of non-fatal suicide attempts among females and higher rates of suicide deaths among males [56,57,58]—often termed the “gender paradox” of suicidal behavior.

The relatively balanced gender distribution in our sample may reflect characteristics of community mental health populations, where males may be more likely to present following serious attempts compared to general population samples but also regional or cultural factors influencing help-seeking behavior in Northern Italy. Furthermore, for the specific age range studied (16–29), gender differences in attempt rates may be less pronounced than in older adults.

Future research with larger samples should examine gender-specific patterns in attempt methods, diagnostic profiles, and treatment outcomes in adolescents and young adults.

## 4. Discussion

This study provides a detailed characterization of individuals presenting to CPS in the aftermath of a suicide attempt, highlighting clinically relevant diagnostic differences in age of onset and associated psychosocial risk profiles. Unlike studies conducted in specialized academic settings, our sample reflects the real-world caseload of Italian community psychiatric services, offering valuable insights for service planning and early intervention strategies.

The finding that personality disorders are associated with an earlier age at first suicide attempt aligns with the previous literature, yet the present study strengthens this evidence within an Italian CPS context. The robustness of the association across multiple statistical approaches—including Welch’s ANOVA and Kruskal-Wallis tests—supports the stability of this effect despite group-size imbalance.

The early emergence of suicidal behavior in individuals with personality disorders underscores the need for enhanced detection of emotional dysregulation and interpersonal instability in primary care and youth mental health services. By contrast, patients with schizophrenia spectrum disorders attempted suicide at significantly later ages, consistent with trajectories typically associated with psychosis onset, illness burden, and cumulative stressors.

The contribution of this study lies in demonstrating how diagnostic patterns of suicidality manifest within a decentralized, community-based system. The CPS model, unlike hospital-based networks, provides ongoing non-specialist psychiatric care, crisis intervention, and longitudinal support. Understanding diagnostic patterns in this environment can inform triage priorities, resource allocation, and the development of targeted suicide-prevention pathways.

### 4.1. Disorder-Specific Trajectories

Our most theoretically significant finding is the differential age at first suicide attempt across diagnostic categories. Patients with personality disorders attempted suicide approximately 5 years earlier than those with schizophrenia spectrum disorders. This pattern reflects the distinct developmental trajectories and natural histories of these conditions.

Personality pathology, particularly borderline personality disorder, often emerges during mid-to-late adolescence [28]. Core features including emotional dysregulation, identity disturbance, intense and unstable relationships, and impulsivity manifest during the teenage years—a developmental period already characterized by heightened emotional intensity, identity exploration, and interpersonal sensitivity. The convergence of normative developmental challenges with emerging personality pathology creates a particularly high-risk context for suicidal behavior.

Research supports the validity and clinical utility of diagnosing personality disorders in adolescence when diagnostic criteria are met [59]. Glenn and Klonsky [60] demonstrated good reliability and validity of BPD diagnosis in hospitalized adolescents, while Paris [28,61] argued that recognizing personality pathology in adolescence enables early intervention with evidence-based treatments such as Dialectical Behavior Therapy (DBT) [62].

In our sample, patients with personality disorders attempted suicide at a mean age of 18.70 years—consistent with the mid-to-late adolescent onset of these conditions. Suicidal behavior in this population often occurs in the context of interpersonal crises, perceived abandonment, or intense affective states, reflecting the core psychopathology of emotional dysregulation and interpersonal instability.

In contrast, schizophrenia spectrum disorders typically have a later age of onset, with first psychotic episodes most commonly occurring in late adolescence to early adulthood (late teens to mid-twenties for males, slightly later for females). In our sample, patients with schizophrenia spectrum disorders attempted suicide at a mean age of 23.64 years—significantly later than those with personality disorders and consistent with the typical age of illness onset.

Suicidal behavior in schizophrenia often emerges during the early phases of illness, particularly following initial diagnosis, during periods of active psychosis, or in response to insight into the chronic nature of the condition. Factors contributing to suicide risk in schizophrenia include command hallucinations, persecutory delusions, depression, hopelessness about prognosis, and social isolation [63].

The later age at first attempt in our schizophrenia spectrum group likely reflects the later developmental onset of psychotic disorders. However, it is important to note that this does not imply lower overall risk. Lifetime suicide rates in schizophrenia are substantial (5–13%), with the highest risk in the first few years following diagnosis.

These developmental differences have important implications for targeted prevention strategies:

- For personality disorders: Prevention and early intervention efforts should target mid-to-late adolescence (ages 15–20), focusing on identifying youth with emerging emotional dysregulation, self-harm, and interpersonal difficulties. School-based screening, training of school counselors and pediatricians, and accessible DBT programs for adolescents may be particularly valuable [64].
- For schizophrenia spectrum disorders: Prevention efforts should target the late adolescent to early adult period (ages 20–25), focusing on early detection of prodromal symptoms, rapid access to first-episode psychosis programs, and intensive support during the critical period following diagnosis [65]. Suicide risk assessment should be integrated into all early psychosis interventions.
- For mood disorders: Although our sample size was small (*n* = 5), patients with mood disorders showed an intermediate pattern (M = 19.60 years), suggesting that prevention efforts should span mid-adolescence through early adulthood, with attention to both early-onset and later-onset depression and bipolar disorder [66].

### 4.2. Treatment Status and Pathways to Care

A striking finding was that nearly 60% of participants were not engaged in mental health treatment at the time of their index suicide attempt. This underscores the importance of prevention efforts upstream of the mental health system. Strategies should include universal school-based mental health education and screening programs [67,68], training of “gatekeepers” (teachers, coaches, pediatricians, and parents) to recognize warning signs, public awareness campaigns to reduce stigma and promote help-seeking [69], accessible, low-barrier mental health services for youth (e.g., school-based clinics, drop-in centers, and telehealth options)**.** In light of this, access to mental healthcare should be made easier and with less bureaucracy for young people experiencing subclinical symptoms or early signs of distress, before escalation to suicidal crisis. Hodgekins et al. [70] found that youth mental health services with flexible referral pathways and youth-friendly environments improved access for at-risk adolescents.

However, it is also important to note that 37% of participants in our study attempted suicide despite being in active treatment at the CPS. This finding highlights the challenges of managing acute suicide risk even within the context of ongoing psychiatric care. Suicide attempts in these patients could reflect the limitations of current interventions for certain high-risk populations (e.g., treatment-resistant conditions) but can also be a red flag for insufficient treatment intensity for critical conditions or poor adherence to treatment coming from a weak therapeutic alliance. Furthermore, we must not forget that acute stressors or crises can precipitate stable conditions. These cases underscore the need for evidence-based, intensive interventions specifically targeting suicidal behavior, such as Dialectical Behavior Therapy (DBT) [71], Cognitive Therapy for Suicide Prevention [72], and Mentalization-Based Therapy [73].

### 4.3. Methods of Suicide Attempt and Lethality

Medication overdose was the predominant method of suicide attempt in our sample (54.8%), consistent with international data showing that self-poisoning is the most common method of non-fatal suicide attempts, particularly among youth and females. The accessibility of medications—both prescription psychotropics and over-the-counter analgesics—contributes to the high frequency of this method.

From a prevention standpoint, this finding suggests several strategies: For example, limiting pack sizes of over-the-counter analgesics (particularly paracetamol/acetaminophen) has been shown to reduce overdose deaths in countries where implemented [74]. Also, clinicians treating high-risk youth should prescribe smaller quantities of potentially lethal medications and consider safer alternatives where possible. Specifically for youths, who often live with their families of origin, educating families about safe storage and disposal of medications is highly recommended.

However, it is important to note that more lethal methods were also represented in our sample, including hanging/suffocation (14.3%), jumping from height (11.9%), and train/vehicle impact (4.8%). The presence of high-lethality methods indicates serious suicidal intent and elevated risk of future fatal attempts in some participants. These cases require intensive intervention and close monitoring.

Gender differences in method choice are well documented in the literature, with males more likely to use high-lethality methods (firearms and hanging) and females more likely to use self-poisoning. Our sample size precluded robust statistical analysis of gender differences in method choice.

### 4.4. Comorbidity: Substance Use and Mood Symptoms

Among participants with available data, 43.2% had documented substance misuse problems. This high rate is consistent with the extensive literature documenting the strong association between substance use disorders and suicidal behavior in youth. Substance use increases suicide risk through multiple mechanisms. One of them is acute intoxication and it impairs judgment, increases impulsivity, and reduces inhibitions against suicidal behavior. Also, chronic use is problematic as it exacerbates underlying psychiatric symptoms (depression, anxiety, and psychosis). Withdrawal states can precipitate mood disturbances and suicidal ideation.

Substance use disorders are associated with psychosocial stressors (academic failure, legal problems, and family conflict) that independently increase risk. The high comorbidity between psychiatric disorders and substance use in our sample underscores the need for integrated treatment approaches that address both conditions simultaneously. Traditional sequential models (treating primary psychiatric disorder first, then addressing substance use) are often ineffective. Instead, evidence-based integrated interventions such as DBT (which addresses both emotional dysregulation and substance use) may be particularly valuable for this population.

Additionally, 38.1% of participants had documented mood symptoms at some point during treatment (though missing data were substantial for this variable). The presence of depressive symptoms substantially elevates suicide risk across diagnostic categories. Comorbidity between depression and anxiety, or depression and other conditions, confers particularly high risk [75]. This finding reinforces the importance of comprehensive assessment and treatment of mood symptoms in all youth presenting with suicidal behavior, regardless of primary diagnosis.

### 4.5. The Gender Paradox in Suicidal Behavior

Our sample showed a relatively balanced gender distribution (53.2% female, 46.8% male), which is somewhat surprising given the well-documented “gender paradox” of suicidal behavior: females have higher rates of non-fatal suicide attempts, while males have higher rates of suicide deaths. This paradox is typically attributed to gender differences in method choice, with males more likely to use highly lethal methods (firearms and hanging) and females more likely to use less immediately lethal methods (self-poisoning).

Several factors may explain the relatively balanced gender distribution in our community mental health sample:

1. Clinical Population vs. General Population: Our sample comprised individuals referred to psychiatric services following suicide attempts. Males who attempt suicide may be more likely to use lethal methods, resulting in death rather than survival and referral to outpatient services. Consequently, community mental health samples may show more balanced gender ratios than general population epidemiological studies.
2. Severity and Intent: Males presenting to services following attempts may represent a selected subgroup with particularly serious intent or high-lethality attempts, whereas the broader population of male attempters (including those with lower intent or lethality) may be less likely to access services.
3. Age Range: The 16–29 age range studied may show different gender patterns than older adult populations. Some research suggests that gender differences in attempt rates are most pronounced in adolescence and narrow somewhat in young adulthood [45].
4. Regional and Cultural Factors: Patterns of help-seeking behavior and suicide attempt rates may vary across regions and cultures. Our Northern Italian sample may reflect local patterns that differ from international norms.

Regardless of the explanation, the substantial representation of males in our sample (46.8%) is clinically significant. Males who attempt suicide are at particularly high risk of future fatal attempts, and engaging this population in ongoing treatment is a critical challenge. Services should develop male-friendly intervention approaches, address barriers to help-seeking among young men, and provide intensive follow-up for male attempters.

### 4.6. Clinical and Public Health Implications

Our findings have several important implications for clinical practice and public health policy.

#### 4.6.1. Developmentally Informed Prevention Strategies

Prevention efforts should be tailored to the developmental timing and natural history of specific psychiatric disorders. For mid-to-late adolescence (ages 15–20) interventions should focus on identifying emerging personality pathology, emotional dysregulation, self-harm, and interpersonal difficulties. Moreover, implementation of school-based screening, gatekeepers training, and accessible DBT-informed interventions should be taken into consideration. For the late adolescence to early adulthood (ages 20–25) phase, preventive strategies should target early detection of psychotic prodrome, rapid access to first-episode psychosis programs, and intensive support following diagnosis of schizophrenia spectrum disorders. Across the entire 16–29 age range, vigilance for mood disorders, anxiety disorders, and substance use disorders, which can emerge at varying ages, must be held high.

#### 4.6.2. Integration of Suicide Risk Assessment into Routine Care

All youth presenting to mental health services should receive comprehensive suicide risk assessment, regardless of presenting complaint. Assessment should include current suicidal ideation, intent, and plans; history of prior attempts and self-harm; psychiatric diagnosis and symptom severity; substance use; recent psychosocial stressors and interpersonal crises; access to lethal means; protective factors (reasons for living, social support, and treatment engagement).

Risk assessment should be repeated regularly, particularly during high-risk periods (e.g., treatment transitions, medication changes, and psychosocial crises).

#### 4.6.3. Evidence-Based Psychotherapeutic Interventions

Youth at high risk for suicidal behavior should receive evidence-based psychotherapeutic interventions specifically targeting suicidality. Dialectical Behavior Therapy (DBT) shows strong evidence for reducing suicide attempts and self-harm in adolescents and adults with BPD and emotional dysregulation. Cognitive Therapy for Suicide Prevention (CT-SP) demonstrated efficacy in reducing repeat attempts [76]. Mentalization-Based Therapy (MBT) is a psychodynamically oriented psychotherapy and is effective for adolescents and adults with BPD [73]. Family-based interventions are important for adolescents, given the central role of family relationships in youth development.

#### 4.6.4. Pharmacological Considerations

Pharmacological treatment should be guided by primary psychiatric diagnosis, with attention to suicide risk. Lithium holds strong evidence for suicide prevention in mood disorders [77,78], though monitoring requirements may limit use in some youth populations. Clozapine has proven effective in reducing suicidal behavior in schizophrenia [79], though it is reserved for treatment-resistant cases due to side effect profile. For antidepressants’ use, careful risk-benefit assessment is required in youth, given FDA black box warning about increased suicidal ideation in pediatric populations [80]. Close monitoring during initiation and dose changes is essential. Esketamine has provided rapid reduction in suicidal ideation in treatment-resistant depression [81], though long-term data in youth are limited.

Importantly, pharmacotherapy alone is insufficient for high-risk youth; it should be combined with psychotherapy and psychosocial interventions [82].

### 4.7. Strengths and Limitations

#### 4.7.1. Strengths

Our study has several strengths:

1. Real-World Clinical Population: Unlike many studies conducted in specialized academic centers, our data come from a community psychiatric service serving a defined catchment area. This enhances generalizability to typical clinical practice settings.

2. Comprehensive Clinical Characterization: We provide detailed information on diagnoses, treatment status, methods of attempt, substance use, and other clinically relevant variables, offering a multifaceted picture of youth suicide attempters.

3. Developmental Focus: Our focus on the 16–29 age range captures the critical transition from adolescence through emerging adulthood—a period of heightened vulnerability and developmental change.

4. Disorder-Specific Analysis: Our examination of age at first attempt across diagnostic categories provides novel insights into the developmental psychopathology of suicidal behavior and has direct implications for targeted prevention.

5. Italian Community Context: We contribute to the limited literature on youth suicidal behavior in Italian community psychiatric settings, providing data relevant to service planning and policy in this context.

#### 4.7.2. Limitations

The present study should be interpreted in light of several limitations. First, the retrospective design, relying on electronic clinical records, inevitably exposes the findings to incomplete or inconsistently recorded information; although sensitivity analyses using pairwise deletion, listwise deletion, and multiple imputation yielded comparable results, the possibility of residual bias cannot be excluded. The substantial proportion of missing data for several variables—such as family psychiatric history, mood symptoms, and educational attainment—further constrains the interpretability of these domains. Moreover, the study lacks longitudinal follow-up, limiting inference to cross-sectional patterns observed at the time of the index suicide attempt; for this reason, any suggestion of developmental or clinical “trajectories” cannot be supported within the current design.

Diagnostic considerations also warrant caution: psychiatric diagnoses were derived from routine ICD-10 codes assigned in clinical practice, without the benefit of structured diagnostic interviews, and were often broad or nonspecific. This is particularly relevant for personality disorders, where only a minority of participants had a documented borderline personality disorder subtype despite clinical features suggestive of emotional dysregulation in the wider F60 group. The statistical analyses were further challenged by markedly unbalanced diagnostic groups, especially in the ANOVA comparing age at first attempt, where the small size of the mood disorder subgroup limits the robustness of between-group comparisons despite the consistency of results across Welch’s ANOVA and non-parametric tests.

Additionally, the cross-sectional snapshot provided here does not allow examination of recurrence risk, treatment response, or long-term psychosocial outcomes, nor does it permit comparison with youth presenting to CPS for reasons other than a suicide attempt, thereby restricting the ability to isolate suicide-specific risk factors. Generalizability is limited by the single-site nature of the sample and by the distinct organizational features of Italy’s community-based psychiatric system, which may not be representative of mental-health settings in other national contexts. Finally, several clinically relevant domains—such as severity and lethality of attempts, detailed psychiatric comorbidity, trauma exposure, bullying, family dynamics, and treatment adherence—were insufficiently documented, precluding a more comprehensive characterization of risk profiles.

#### 4.7.3. Recommendations for Future Research

Future research should seek to overcome the limitations inherent in the present study by adopting prospective and longitudinal designs capable of following youths from the first suicidal episode through sustained clinical trajectories and long-term outcomes. Larger, multi-site samples are needed to ensure adequate statistical power, particularly for subgroup analyses, and should ideally incorporate structured diagnostic interviews to enhance diagnostic reliability and validity. A more rigorous and systematic characterization of suicide attempts—including objective ratings of severity, lethality, and medical consequences—would substantially strengthen the clinical interpretability of findings.

Equally essential is the comprehensive assessment of psychiatric comorbidity, with particular attention to substance use disorders, anxiety disorders, and trauma-related conditions, domains that are often underdocumented in routine clinical settings but critically relevant to suicide risk. Future studies should also integrate detailed psychosocial evaluations encompassing family functioning, adverse experiences, bullying, social support, and contextual stressors, thereby enabling a more nuanced understanding of vulnerability pathways. The inclusion of appropriate comparison groups—such as psychiatric controls without a history of suicide attempts or community controls—would further clarify factors that distinguish attempters from non-attempters.

Longitudinal investigations examining treatment response, the predictors of repeated attempts, and the emergence of protective and resilience factors in high-risk youth would provide crucial guidance for preventive interventions. Moreover, gender-specific analyses of attempt methods, diagnostic correlates, and treatment outcomes may illuminate clinically important divergences that remain insufficiently explored.

Although missing data were relatively limited in the present study, pairwise deletion may theoretically introduce bias; however, the consistency of findings across listwise deletion and multiple-imputation sensitivity analyses mitigates concerns regarding the impact of missingness on the conclusions drawn.

## 5. Conclusions

This retrospective study of 63 adolescents and young adults who attempted suicide and were referred to a community psychiatric service in Northern Italy provides important insights into the developmental psychopathology of youth suicidal behavior. Personality disorders, particularly borderline personality disorder, were the most common diagnosis (50.9%), followed by schizophrenia spectrum disorders (26.4%) and mood disorders (15.1%). Age at index suicide attempt differed significantly across diagnostic categories, with patients affected by personality disorders attempting suicide approximately 5 years earlier (M = 18.70 years) than those with schizophrenia spectrum disorders (M = 23.64 years). This pattern reflects the distinct developmental trajectories of these conditions. Medication overdose was the predominant method of attempt (54.8%), though more lethal methods were also represented, indicating serious intent in a substantial subgroup. A particularly relevant datum is that nearly 60% of participants were not engaged in mental health treatment at the time of their index attempt, highlighting critical gaps in pathways to care and the need for upstream prevention efforts. On the other hand, for those already under treatment, it is of paramount importance to promote integrated treatment of both psychiatric and substance use disorders, given the high rate of substance misuse (43.2% among those with data). As far as prevention is concerned, strategies should be tailored to the developmental timing of specific psychiatric disorders, with efforts targeting mid-to-late adolescence for personality disorders and late adolescence to early adulthood for schizophrenia spectrum disorders. Furthermore, routine suicide risk assessment should be integrated into all youth mental health services, regardless of presenting complaint. Referring to treatment, evidence-based psychotherapeutic interventions specifically targeting suicidality (DBT, CT-SP, and MBT) should be made widely accessible to high-risk youth.

- Systems-level interventions are needed to facilitate earlier access to care before youth reach suicidal crisis, including school-based services, telehealth options, and youth-friendly service models.
- Means restriction strategies (safe medication storage, and reduced pack sizes of analgesics) should be implemented at both individual and policy levels.

While our study has important limitations—including retrospective design, small sample size, missing data, and lack of longitudinal follow-up—it contributes valuable real-world data from a community psychiatric setting. Our findings support the development of developmentally informed, disorder-specific prevention strategies and early intervention programs tailored to the natural course and psychopathology of specific psychiatric conditions affecting youth.

Ultimately, reducing youth suicide requires a comprehensive, multi-level approach encompassing universal prevention (mental health education, and stigma reduction), selective prevention (targeting high-risk groups), indicated prevention (early intervention for symptomatic youth), and evidence-based treatment for those who have already attempted suicide. Our findings underscore the critical importance of early identification, accessible pathways to care, and intensive, evidence-based interventions for this vulnerable population.

## Figures and Tables

**Table 1:** Robustness checks and effect sizes for group comparisons.

Test	Statistic	*p*-Value	Effect Size	Interpretation
ANOVA (Omnibus)	F = 14.82	*p* < 0.001	η^2^ = 0.32	Large effect
Welch ANOVA	F_w = 13.77	*p* < 0.001	—	Consistent with ANOVA
Kruskal-Wallis	H = 12.91	*p* < 0.001	—	Consistent (non-parametric)
PD vs SSD	Tukey	*p* < 0.001	d = 1.54	Large effect
PD vs MD	Tukey	*p* = 0.21	d = 0.35	Small effect
MD vs SSD	Tukey	*p* = 0.004	d = 1.21	Large effect

**Table 2:** Demographic and clinical characteristics of the sample (*N* = 63).

Characteristic	*n* (%) or M (SD)
**Demographics**	
Age at index attempt (years) ^a^	20.20 (3.46), range 16–29
**Gender**	
Female	33 (52.4%)
Male	29 (46.0%)
Missing	1 (1.6%)
**Primary Psychiatric Diagnosis (ICD-10)** ^b^	
Personality Disorders (F6×)	27 (50.9%)
- F60.31 Borderline PD	7 (13.2%)
- F60/F60.9 PD unspecified	16 (30.2%)
- Other F6 codes	4 (7.5%)
Schizophrenia Spectrum (F2×)	14 (26.4%)
Mood Disorders (F3×)	8 (15.1%)
Anxiety/Stress-Related (F4×)	3 (5.7%)
Developmental Disorders (F8×)	1 (1.9%)
**Clinical Features**	
Mood symptoms present ^c^	24 (38.1%)
Substance misuse ^d^	16/37 (43.2%)
Family psychiatric history ^e^	4 (6.3%)
**Index Suicide Attempt Characteristics**	
Method of attempt ^f^	
Medication overdose	23 (54.8%)
Hanging/suffocation	6 (14.3%)
Jumping from height	5 (11.9%)
Cutting/stabbing	3 (7.1%)
Train/vehicle impact	2 (4.8%)
Poisoning (non-medication)	2 (4.8%)
Other	1 (2.4%)
Repeat attempts ^g^	11/41 (26.8%)
**Treatment Status at Time of Attempt** ^h^	
First access to services	27/46 (58.7%)
During active treatment	17/46 (37.0%)
In history	2/46 (4.3%)
**Pharmacological Treatment at Time of Attempt** ^i^	
None	13/33 (39.4%)
Antipsychotic	9/33 (27.3%)
Polypharmacy	6/33 (18.2%)
Antidepressant	3/33 (9.1%)
Mood stabilizer	2/33 (6.1%)

**Notes:** ^a^ *n* = 40 with complete age data. ^b^ *n* = 53 with primary diagnosis (84.1% of sample); percentages calculated among those with diagnosis. ^c^ 61.9% missing data (*n* = 39). ^d^ Data available for *n* = 37 (58.7%); percentage among those with data. ^e^ 93.7% missing data (*n* = 59). ^f^ Data available for *n* = 42 (66.7%); percentages among those with data. ^g^ Data available for *n* = 41 (65.1%). ^h^ Data available for *n* = 46 (73.0%). ^i^ Data available for *n* = 33 (52.4%).

## Data Availability

The original contributions presented in this study are included in the article. Further inquiries can be directed to the corresponding author.
